# Mutations of the mitochondrial carrier translocase channel subunit TIM22 cause early-onset mitochondrial myopathy

**DOI:** 10.1093/hmg/ddy305

**Published:** 2018-08-24

**Authors:** David Pacheu-Grau, Sylvie Callegari, Sonia Emperador, Kyle Thompson, Abhishek Aich, Sarah E Topol, Emily G Spencer, Robert McFarland, Eduardo Ruiz-Pesini, Ali Torkamani, Robert W Taylor, Julio Montoya, Peter Rehling

**Affiliations:** 1 Department of Cellular Biochemistry, University Medical Center Göttingen, Göttingen, D-37073, Germany; 2 Departamento de Bioquímica y Biología Molecular y Celular, Universidad de Zaragoza-CIBER de Enfermedades Raras (CIBERER)-Instituto de Investigación Sanitaria de Aragón (IIS Aragón), Zaragoza, 50013, Spain; 3 Max-Planck Institute for Biophysical Chemistry, D-37077, Göttingen, Germany; 4 Wellcome Centre for Mitochondrial Research, Institute of Neuroscience, Newcastle University, Newcastle upon Tyne, NE2 4HH, United Kingdom; 5 The Scripps Translational Science Institute, The Scripps Research Institute, La Jolla, CA 92037, United States; 6 Department of Integrative Structural and Computational Biology, The Scripps Research Institute, La Jolla, CA 92037, United States

## Abstract

Protein import into mitochondria is facilitated by translocases within the outer and the inner mitochondrial membranes that are dedicated to a highly specific subset of client proteins. The mitochondrial carrier translocase (TIM22 complex) inserts multispanning proteins, such as mitochondrial metabolite carriers and translocase subunits (TIM23, TIM17A/B and TIM22), into the inner mitochondrial membrane. Both types of substrates are essential for mitochondrial metabolic function and biogenesis. Here, we report on a subject, diagnosed at 1.5 years, with a neuromuscular presentation, comprising hypotonia, gastroesophageal reflux disease and persistently elevated serum and Cerebrospinal fluid lactate (CSF). Patient fibroblasts displayed reduced oxidative capacity and altered mitochondrial morphology. Using trans-mitochondrial cybrid cell lines, we excluded a candidate variant in mitochondrial DNA as causative of these effects. Whole-exome sequencing identified compound heterozygous variants in the *TIM22* gene (NM_013337), resulting in premature truncation in one allele (p.Tyr25Ter) and a point mutation in a conserved residue (p.Val33Leu), within the intermembrane space region, of the TIM22 protein in the second allele. Although mRNA transcripts of *TIM22* were elevated, biochemical analyses revealed lower levels of TIM22 protein and an even greater deficiency of TIM22 complex formation. In agreement with a defect in carrier translocase function, carrier protein amounts in the inner membrane were found to be reduced. This is the first report of pathogenic variants in the TIM22 pore-forming subunit of the carrier translocase affecting the biogenesis of inner mitochondrial membrane proteins critical for metabolite exchange.

## Introduction

Mitochondria represent metabolic core units and signaling hubs of eukaryotic cells. For their central role in energy production through oxidative phosphorylation, the inner membrane has to maintain a proton gradient (ΔpH) that drives Adenosine triphosphate (ATP) production in the matrix. Hence, the transport of metabolites into and out of mitochondria is mediated by dedicated transport systems that utilize the proton gradient as a driving force, but maintain the ΔpH. This is true for metabolite carriers and also for protein transport machineries.

Mitochondria import the vast majority of proteins from the cytosol, while only 13 proteins are mitochondrial-encoded. The translocase of the outer mitochondrial membrane (TOM complex) represents the general entry port into mitochondria for precursor proteins. Upon passage through the TOM complex, precursors are segregated to engage with dedicated translocases in the outer membrane, the intermembrane space (IMS) and the inner membrane in a signal-specific manner (1–4). The carrier translocase (TIM22 complex) mediates the transport of carrier proteins for inner membrane insertion. These precursors utilize internal targeting signals for transport (5,6). In addition to the six transmembrane span containing carrier proteins, TIM22 cargoes also include the four transmembrane span containing channel-forming subunits of the TIM23 complex (TIM23 and TIM17A/B) and the TIM22 protein itself (7–12).

**Figure 1:**
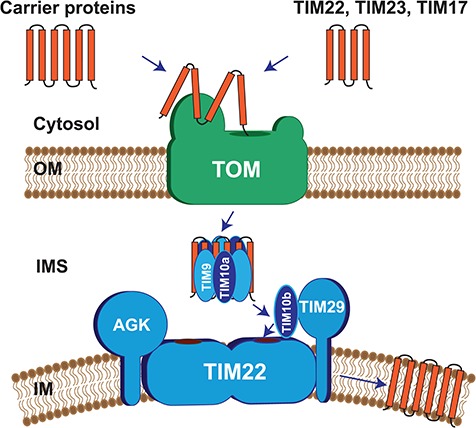
Composition of human TIM22 complex and mechanism of action. The TIM22 complex is comprised of the central twin-pore forming unit TIM22, in addition to TIM10B and the metazoan specific subunits, TIM29 and AGK. A soluble hexameric ring, made up of TIM9 and TIM10A and present in the IMS, guides precursors (carrier proteins or TIM22, TIM23, TIM17A/B) from the TOM complex to the TIM22 complex. Upon docking to the TIM22 complex, the precursor is released into the TIM22 channel and its insertion into the membrane is driven by the mitochondrial membrane potential Δψ.

The TIM22 complex is comprised of a central twin-pore forming unit, made up of two channels formed by the TIM22 protein (13,14). Additional components include the conserved TIM10B and the metazoan-specific subunits, TIM29 and acylglycerol kinase (AGK) (7–10,15). Together, these proteins constitute a complex of ∼440 kDa. Both TIM29 and AGK are required for the stability and import competence of the translocase (7,8). Two conserved soluble hexameric rings, made up of TIM9 and TIM10A as well as TIM8A and TIM13, respectively, reside in the mitochondrial IMS and are responsible for guiding the precursor from the TOM complex to the TIM22 complex (16–19). Upon docking to the TIM22 complex, the precursor is released into the TIM22 channel and its insertion into the membrane is driven by the mitochondrial membrane potential Δψ (13,20) (Fig. 1).

The function of the TIM22 complex is conserved from yeast to human and loss of TIM22 in yeast is lethal (14,21). This is not surprising, given the role of TIM22 for not only the insertion of metabolite carrier proteins, but also for the biogenesis of the TIM23 complex, which is responsible for the import of precursors with N-terminal presequences that represent ∼60% of the mitochondrial proteome (5,22). As such, disease-causing mutations in components of the carrier translocase are extremely rare (23). Recently, AGK, which had been implicated in Sengers syndrome, was identified as a component of the TIM22 complex. AGK is a bifunctional protein. In addition to being a carrier translocase subunit, AGK functions as lipid kinase in mitochondrial lipid metabolism. Its role in the carrier translocase is apparently independent of its kinase function and could explain the metabolic defects observed in patients with Sengers syndrome (9,10). Accordingly, both functions contribute to the observed mitochondrial pathology. Moreover, pathogenic variants have been reported in TIM8A, associated with deafness-dystonia-optic neuronopathy syndrome, also known as Mohr–Tranebjaerg syndrome (24,25). However, the substrate spectrum and the role of TIM8A in protein transport in human remain poorly defined.

Here we report a patient with gastroesophageal reflux disease, hypotonia and persistently elevated lactate levels with biochemical evidence of mitochondrial dysfunction in fibroblasts. Whole-exome sequencing identified compound heterozygous variants in the *TIM22* gene, encoding the TIM22 channel protein of the carrier translocase. Despite elevated expression of *TIM22* mRNA, biochemical analyses demonstrate severely compromised levels of the TIM22 protein and, concomitantly, of the TIM22 complex. We found a reduced amount of tested carrier proteins, which are imported by the TIM22 complex, in the inner mitochondrial membrane. Our analyses link mutations in the core subunit of the carrier translocase to the complex pathology of a human disorder through a defect in the transport of metabolite carriers.

**Figure 2:**
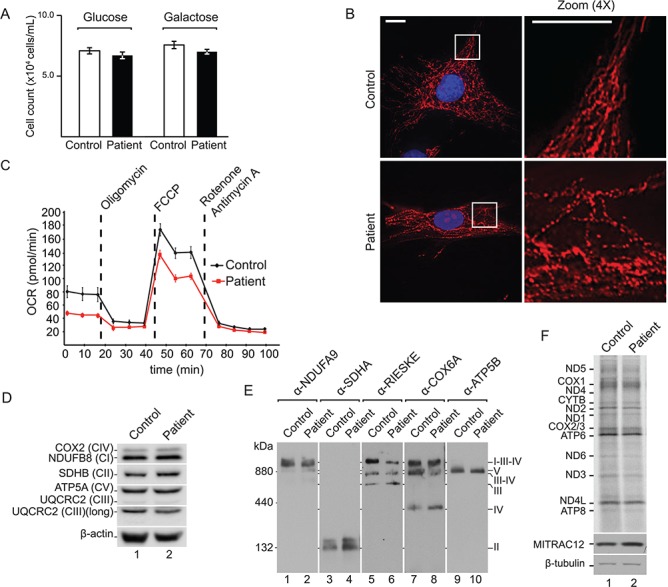
Patient fibroblasts exhibit reduced mitochondrial fitness. (**A**) Cell counts of control and patient cells after 3 days of growth on glucose or galactose (Standard error of the mean (SEM), *n* = 3). (**B**) Mitochondrial network of a representative control and patient fibroblast cell, stained with MitoTracker and DAPI. Scale bar = 15 μm. (**C**) Real-time respirometry of patient and control cells; OCR. (**D**) SDS-PAGE and western blot analysis of OXPHOS subunits with indicated antibodies in control and patient fibroblast lysates using β-actin as loading control. (**E**) blue native polyacrylamide gel electrophoresis (BN-PAGE) and western blot analysis of OXPHOS complexes in solubilized mitochondria from control and patient fibroblasts using indicated antibodies**.** (**F**) *In vivo* labelling of mitochondrial translation products in control and patient immortalized cells. Samples were analysed by SDS-PAGE, digital autoradiography and western blotting.

## Results

### Case presentation

We investigated the molecular basis of a presumed mitochondrial disorder in a 10-year-old British female of mixed ethnic background, previously reported in the literature (26). She is the third child of a healthy, non-consanguineous parents, with no familial history of neurological disease apart from epilepsy in a maternal aunt. Intrauterine growth retardation was observed at 32 weeks of pregnancy. At birth, she had a low birth weight (2.33 kg) and below-average height (45 cm) and showed reduced spontaneous movements and hypotonia. The subject presented with feeding difficulties, gastroesophageal reflux with projectile vomiting and acetabular dysplasia. Brain magnetic resonance imaging at 8 months revealed a delay in white matter myelination. She remained proportionately small (height, weight and head circumference all below the 0.4^th^ centile) and weak throughout childhood. Biochemical analyses revealed increased lactate (4.2 mmol/L; normal controls, 0.5–2.2 mmol/L) and creatine kinase (282 U/L; normal controls, 100–190 U/L) levels in plasma and she underwent a diagnostic muscle biopsy on suspicion of mitochondrial disease. This identified decreased activities of respiratory chain complexes I, III and IV with sparing of complex II activity (26). Having excluded mitochondrial DNA (mtDNA) rearrangements and a quantitative loss of mtDNA copy number, full mtDNA sequencing revealed a rare homoplasmic m.5514A>G mt-tRNA^Trp^ variant not present in >3000 human mtDNA control sequences (26). The m.5514A>G transition affects an A-U base pair in the acceptor stem of mt-tRNA^Trp^, however, this position in the transfer RNA (tRNA) molecule shows poor evolutionary conservation (26). The m.5514A>G variant was homoplasmic in the blood from her clinically unaffected mother, prompting further studies to assess pathogenicity and the possible implications of this rare mtDNA variant at a cellular level.

### Assessment of mitochondrial function in patient fibroblasts

To assess the cellular effects that underlie the mitochondrial phenotype, primary fibroblasts of the patient and age-matched controls were obtained. First, to assay the capacity of the oxidative phosphorylation (OXPHOS) system in patient cells, we measured the growth rate of cells in glucose and galactose medium for 3 days. In glucose medium, cells depend on glycolysis, whereas in galactose medium, cells depend almost exclusively on OXPHOS to produce the required amount of ATP and maintain a physiological proliferation rate (27). Surprisingly, patient-derived fibroblasts showed a similar growth rate compared to those of the control (Fig. 2A). When the mitochondrial network was analysed in fibroblast cells by fluorescence microscopy in cells stained with MitoTracker, patient cells displayed a partially fragmented mitochondrial network when compared to controls (Fig. 2B). Next, we assessed respiratory chain function—reported to be abnormal in patient muscle—in fibroblasts, assessing oxygen consumption in intact cells by real-time respirometry. Patient fibroblasts displayed a lower oxygen consumption rate (OCR) during both basal and uncoupled respiration (Fig. 2C). However, these differences were much milder than those reported in other cases of multiple respiratory chain deficiencies, such as the loss of the ribosome assembly factor c7orf30 (leading to a combined complex I, III and IV deficiency) (28), or in cells lacking the complex IV assembly factor COA6 (complex I and IV defects) (29,30). In addition, there was no obvious decrease in the steady-state protein levels of OXPHOS subunits or of assembled complexes (Fig. 2D and 2E). An assessment of the levels of mitochondrial-encoded translation products using [^35^S] methionine showed that the protein synthesis rates were similar in both control and patient cells, indicating no significant mitochondrial translation defect (Fig. 2E), unlike other reported pathological tRNA variants (31,32). Thus, we concluded that, in contrast to skeletal muscle, the patient fibroblasts showed only a mild mitochondrial OXPHOS dysfunction and reduced integrity of the mitochondrial network.

### A nuclear genetic basis of the mitochondrial phenotype

**Figure 3:**
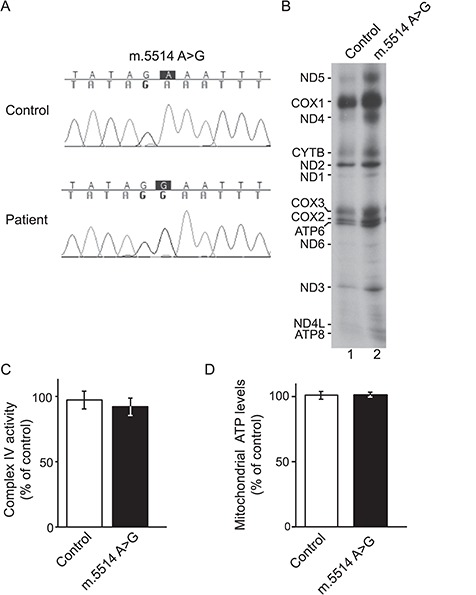
A nuclear genetic basis of the mitochondrial phenotype. (**A**) Sequencing electropherogram showing the presence or absence of the m.5514A>G in A549 cybrid cells. (**B**) *In vivo* labelling of mitochondrial translation products in control and mutant cybrids. Samples were analysed by SDS-PAGE and autoradiography. (**C**) Cytochrome *c* oxidase enzymatic activity, normalized by the enzymatic activity of CS, of mutant cybrid cells relative to control cybrids (Standard deviation (STDEV), *n* = 3). (**D**) Levels of mitochondrial ATP in mutant cybrids compared to control (STDEV, *n* = 6).

To assess whether factors in the nuclear genetic background affect the phenotypic expression of the mitochondrial m.5514A>G variant, we generated transmitochondrial cybrid cells, fusing platelets from the patient and controls with lung carcinoma cells (A549) lacking mtDNA (rho^0^). Sequencing analysis confirmed the presence or absence of the m.5514A>G variant in the obtained cell lines (Fig. 3A) that share an identical nuclear genetic background. Assessment of mitochondrial protein synthesis rates by [^35^S] methionine labelling failed to show decreased mitochondrial translation in the mutant cybrid cells (Fig. 3B). Defects in mitochondrial translation affect the synthesis of core subunits of the OXPHOS system, prompting the measurement of the enzymatic activity of cytochrome *c* oxidase and levels of mitochondrial ATP in mutant cybrid cells. Cells harbouring 100% m.5514A>G showed control levels of complex IV enzyme activity and ATP levels (Fig. 3C and D). Taken together, these analyses demonstrated that a different nuclear genetic environment can complement the mitochondrial OXPHOS phenotype and that the m.5514A>G variant was not responsible for the mitochondrial phenotype.

### Identification of compound heterozygous variants in *TIM22*

**Figure 4:**
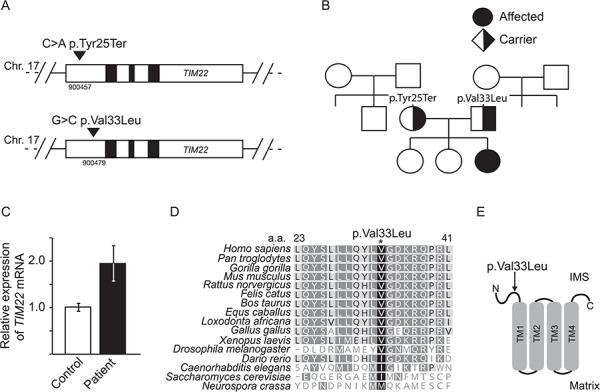
Identification of compound heterozygous variants in *TIM22*. (**A**) New mutations identified in the *TIM22* gene locus. (**B**) Family pedigree showing patient and known unaffected relatives carrying single heterozygous variants. Circle denotes female and square denotes male family members. (**C**) Quantitative PCR of *TIM22* mRNA levels in patient fibroblasts, relative to control fibroblasts (set to 1.0). *TIM22* mRNA levels were normalized to *HPRT* (SEM, *n* = 3). (**D**) Amino acid sequence alignment of the p.Val33Leu containing-segment of TIM22 in indicated organisms. (**E**) Position of variant p.Val33Leu according to the predicted topology of TIM22.

To identify the underlying genetic defect, blood DNA from the patient was subjected to whole-exome sequencing, followed by previously described bioinformatics filtration for candidate coding or splicing variants that segregate in a manner consistent with the segregation of the disease in the family and incidence of the disorder in the general population (33). Inspection of the candidate variant list for mitochondrial genes revealed two heterozygous variants in the *TIM22* gene (NM_013337), encoding the core channel-forming subunit of the carrier translocase of the inner mitochondrial membrane. The identified variants—NM_013337.2:c.74C>A, TIM22:p.Tyr25Ter and NM_013337.2:c.97G>C, TIM22:p.Val33Leu—had not been previously associated with a disease (Fig. 4A). The p.Tyr25Ter and p.Val33Leu variants have been previously observed at population frequencies of ∼0.006% (7 of 115 100 individuals) and ∼0.4% (486 of 116 768 individuals), respectively, among individuals of unknown disease status catalogued in the ExAC database. No homozygous individuals have been previously identified with the p.Tyr25^*^ variant, whereas four individuals of unknown disease status have been reported to carry the p.Val33Leu variant in homozygosity (34). Due to the essential role of TIM22 in mitochondrial protein biogenesis, it is likely that homozygous variants are deleterious. Familial segregation studies confirmed that the unaffected mother carries the heterozygous p.Tyr25^*^ variant and the father is a carrier of the p.Val33Leu variant (Fig. 4B). Quantitative polymerase chain reaction (PCR) measurements of *TIM22* transcripts from the patient fibroblasts showed an increase in *TIM22* mRNA by almost 2-fold, compared to control fibroblasts (Fig. 4C). Therefore, the patient’s phenotype is probably due to a rare genetic event that combines the single effects of both variants. Whereas the p.Tyr25^*^ is definitively pathogenic, predicting loss of TIM22 protein expression, the molecular effect of the p.Val33Leu variant on TIM22 function remains to be determined. Indeed, valine 33 is highly conserved in higher eukaryotes, whereas similar amino acids (like isoleucine or methionine) are found in more evolutionarily distant species like *Saccharomyces cerevisiae* or *Neurospora crassa* (Fig. 4D). According to the predicted topology of the TIM22 protein (35), the position of valine 33 is located in the N-terminal segment, which resides in the IMS before the first transmembrane span (Fig. 4E).

### TIM22 p.Val33Leu destabilizes the carrier translocase and affects metabolite carrier levels

**Figure 5:**
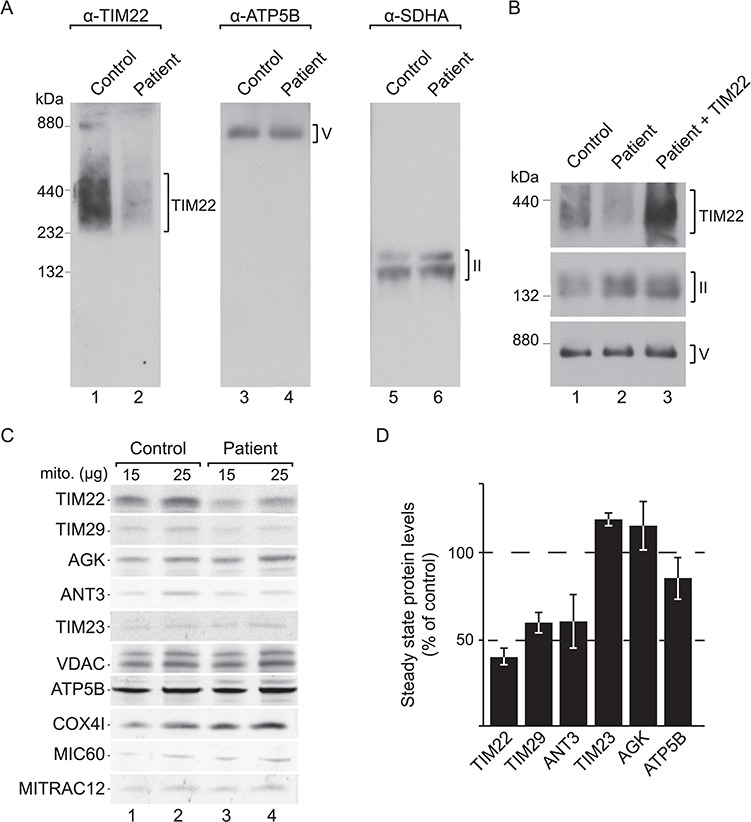
The p.Val33Leu variant destabilizes the carrier translocase and affects metabolite carrier levels. (**A**) Isolated mitochondria from control and patient fibroblasts were solubilized and resolved using BN-PAGE, followed by western blotting and detection using the indicated antibodies. (**B**) Patient immortalized fibroblast cells were electroporated with wild-type TIM22. Mitochondria from these cells and from non-electroporated control and patient cells were isolated, solubilized and analysed by BN-PAGE, followed by western blotting and detection using the indicated antibodies. (**C**) Isolated mitochondria from control and patient fibroblasts were subjected to SDS-PAGE and western blotting. (**D**) Quantification of western blotting signals using infrared secondary antibodies in patient mitochondria compared to control and normalized to the VDAC signal (SEM, *n* = 3).

To determine the effect of TIM22 p.Val33Leu on mitochondrial function, we assessed the integrity of the TIM22 complex. N-Dodecyl-β-maltoside (DDM)-solubilized mitochondrial membranes derived from either control or patient fibroblast were separated by blue native polyacrylamide gel electrophoresis (PAGE) and analysed by western blotting. The human TIM22 complex displays an apparent molecular mass of ∼440 kDa (7,8) and was detected in control mitochondria when using antibodies directed against TIM22. Interestingly, patient mitochondria displayed a drastically reduced TIM22 signal, whereas complex V (ATP5B) and complex II (SDHA) were similar in both samples (Fig. 5A**)**. Re-expression of wild-type TIM22 in patient fibroblasts restored the TIM22 complex (Fig. 5B), indicating that the amount of TIM22 or the Val to Leu exchange affect assembly or stability of the translocase.

The human carrier translocase consists of the channel-forming TIM22, TIM10B and two metazoan-specific components, TIM29 and AGK. To understand if the reduction in fully assembled complex in mutant mitochondria reflects only decreased levels of TIM22, or if other components of the translocase were also affected, we performed western blotting analyses after sodium dodecyl sulfate-polyacrylamide gel electrophoresis (SDS-PAGE). In mitochondria from patient fibroblasts, TIM22 levels were decreased, as well as TIM29, in accordance with previously published data showing that the amounts of these two proteins are interdependent (8). On the contrary, the levels of AGK remained similar in patient mitochondria compared to the control mitochondria, probably due to the second function of this enzyme in lipid metabolism (9,10). In addition, the levels of the carrier translocase transport substrate, ANT3 (ADP/ATP carrier), were clearly decreased in the patient sample. Steady-state amounts of the inner membrane translocase subunit, TIM23 (another known substrate of the TIM22 complex), as well as presequence pathway proteins, such as ATP5B, MIC60 and MITRAC12, remained similar to the control (Fig. 5B). A semi-quantitative assessment of the protein levels showed that at steady-state TIM22 p.Val33Leu and TIM29 were reduced to ∼40% and ∼60% of wild type (WT) level, respectively, in mitochondria from the patient cells. ANT3 levels were also significantly decreased (∼60% of WT) (Fig. 5C). In summary, we demonstrated that the TIM22 p.Val33Leu variant significantly reduced the levels of TIM22 and the structural component TIM29, resulting in a drastic loss of the translocation machinery. Concomitantly, steady-state levels of carrier substrate proteins are reduced in mitochondria. We speculate that the mitochondrial respiratory phenotype and disrupted morphology observed in patient fibroblasts are probably the result of an imbalance of metabolites inside mitochondria, derived from impaired import and insertion of carrier proteins to the inner membrane.

## Discussion

We report here a patient with a neuromuscular presentation of mitochondrial disease and, what we consider may be, the first described pathological variants in TIM22 as a cause of Mendelian mitochondrial disease. In yeast, defects in the Tim22 protein, the channel-forming subunit of the carrier translocase, impairs cell viability. The human TIM22 protein shares 41% similarity with its yeast counterpart (15) and it is likely that its function remains essential in higher eukaryotes. Severe perturbations are expected to result in embryonic lethality and this could explain the lack of known pathological variants in TIM22 identified to date. Therefore, the underlying defect in our patient is the result of a genetic event, combining two rare variants in TIM22, one that generates a premature stop codon (p.Tyr25^*^) and a second variant that produces a single amino acid change (p.Val33Leu). Both genetic variants are present as single heterozygous changes in unaffected parents of the patient, confirming carrier status.

We found that the pVal33Leu mutation of TIM22 disrupts translocase complex formation and compromises levels of metabolite carrier proteins within the inner membrane. Other known substrates of the TIM22 complex are the components of the presequence translocase TIM17A/B and TIM23. Surprisingly, we didn’t observe a decreased level of TIM23 in patient mitochondria. However, both Sengers syndrome patient fibroblasts harbouring *AGK* variants and HEK293 AGK knockout cells, where the 440 kDa TIM22 dissociates into a smaller undefined complex, failed to show significantly reduced import and assembly or decreased levels of TIM23 (10). Recent quantitative analyses of the abundance of mitochondrial proteins in yeast showed that in fully respiring yeast cells (grown on a non-fermentable carbon source), components of the translocases of the inner membrane (TIM22 and TIM23) have relatively low abundance within mitochondria (∼11 400 copies/cell of Tim23 and ∼1050 copies of Tim22), in comparison with, for example, metabolic enzymes (Aco1 ∼152 000 copies/cell), metabolite carriers (Aac1 ∼27 500 copies/cell) or subunits of the respiratory chain (Cox4 ∼91 700 copies/cell) (36). This demonstrates that the import of substrates through the inner membrane is extremely efficient and, therefore, it is probable that the lower steady-state levels of TIM22 complex in patient mitochondria are still capable of importing and maintaining physiological levels of critical cargos. It could also be speculated that there is an additional layer of regulation of the carrier translocase that preferentially supports the biogenesis of certain substrates, such as TIM23 channel components, but this remains to be determined.

Interestingly, protein levels of TIM22 were significantly decreased in patient fibroblasts, despite the fact that *TIM22* mRNA expression was increased by almost 2-fold (reflecting either an up-regulation or a decreased degradation of the mRNA). This suggests that the p.Val33Leu variant destabilizes the protein, either at a structural level or by interfering with complex biogenesis, which is likely responsible for the loss of TIM22 protein. Further analyses of the p.Val33Leu variant are necessary to assess the effect of this variant on the function of TIM22 and on the expression of the disease. Furthermore, the up-regulation of *TIM22* provides evidence of a positive feedback loop, which alters transcription to compensate for protein loss. The existence of a tight regulatory system highlights the critical role TIM22 has for mitochondrial homeostasis. The mechanisms that govern this feedback loop would provide valuable information regarding the regulation of carrier import.

Defects in metabolite carrier import into mitochondria have been associated with other forms of Mendelian mitochondrial disease. Sengers syndrome is a mitochondrial disorder, presenting with congenital cataracts, hypertrophic cardiomyopathy, skeletal myopathy, exercise intolerance, lactic acidosis and delayed motor development. The disease can manifest in childhood or early adult life and has been associated with defects in the AGK (37). In addition to its role in mitochondrial lipid metabolism, AGK has recently been shown to be a component of the TIM22 complex (9,10). However, it is unclear to which extent the different phenotypes observed manifest because of defects in carrier transport, lipid metabolism or both. Interestingly, we observed the same levels of AGK in TIM22 mutant mitochondria, meaning that neither TIM22 nor TIM29 are required for the stability of AGK. Although some of the TIM22 patient’s symptoms may resemble those from Sengers syndrome patients, the molecular defects that we observed are solely associated with a carrier import deficiency and not with complementary lipid metabolism defects through loss of AGK. Moreover, mutations in *DDP1*/*TIM8A* are the cause of Mohr–Tranebjaerg syndrome, also known as deafness-dystonia syndrome. This is a recessive neurodegenerative disorder characterized by progressive sensorineural deafness, cortical blindness, dystonia, dysphagia and paranoia (24). TIM8A forms a hexameric ring, together with TIM13. The presence of mutations in TIM8A may disrupt formation of this complex (25). The yeast Tim8-Tim13 complex has been suggested to participate in the import of Tim23 (38,39). However, import processes differ between yeast and human mitochondria and the TIM22 pathway is the most divergent (23). Therefore, it remains unclear which substrates depend on the TIM8A-TIM13 complex for import and more so, how pathological mutations in TIM8A would affect TIM22 function.

Mutations within single mitochondrial-metabolite carrier proteins have a strong impact on mitochondrial function and many have been associated to disease. For example, dominantly inherited mutations in *SLC25A4* encoding the ADP/ATP carrier AAC1 (ANT1) cause adult-onset autosomal dominant progressive external ophthalmoplegia, associated with multiple mtDNA deletions, whereas recessive *SLC25A4* mutations cause childhood-onset mitochondrial myopathy and cardiomyopathy (40); a recent report has also identified *de novo* dominant *SLC25A4* variants, affecting key residues, causing mtDNA depletion. In addition, mutations in *SLC25A24* (ATP-Mg/Pi carrier) cause Gorlin–Chaudhry–Moss syndrome, a dysmorphic syndrome, characterized by coronal craniosynostosis and severe midface hypoplasia, body and facial hypertrichosis, together with progeroid appearance and mitochondrial dysfunction (41). Therefore, the identification of a novel mutation in TIM22 is a key not only for a better understanding of the mechanisms leading to metabolite carrier import in human mitochondria, but also to dig further into the pathological roles of the loss of carrier proteins in mitochondrial disease.

In summary, the process of identifying the molecular defect underlying the phenotype of this patient nicely illustrates the inherent complexity and heterogeneity associated with mitochondrial diseases. Two very different genomes are involved in the expression of the mitochondrial proteome and therefore, potential variants of both genomes need to be assessed for pathogenicity. In this case, we identified for the first time, compound heterozygous variants in *TIM22* in a patient with salient clinical features of mitochondrial disease. We have shown that these variants exert a molecular impact on the integrity of the carrier translocase, and alter the levels of carrier proteins within mitochondria. This alteration might cause an imbalance of mitochondrial metabolites and lead to a secondary dysfunction of OXPHOS, thus establishing *TIM22* to be the causal gene in this patient.

## Materials and Methods

### Cell culture

Primary fibroblasts were cultured in Dulbecco's modified Eagle's medium (DMEM), supplemented with 10% (v/V) heat-inactivated fetal bovine serum (Biochrom, Berlin, Germany), 2 mM L-glutamine, 1 mM sodium pyruvate and 50 μg/ml uridine and incubated at 37°C with 5% CO_2_. Primary fibroblasts were immortalized using as previously described (42) lung carcinoma A549 cybrids, generated by fusing platelets containing mitochondria and mtDNA with rho^0^ cells, were cultured as previously described (43,44). Growth test experiments were performed by seeding cells directly in medium where glucose was replaced by 0.9 g/L of galactose. Cells were counted after 3 days using a hemocytometer.

For electroporation experiments, immortalized patient fibroblast cells were grown to 70% confluency. Cells were harvested, washed in phosphate-buffered saline (PBS) and resuspended to a density of 5×10^7^ cells/ml in electroporation buffer, provided with the Neon™ transfection system kit (Invitrogen, Germany). Cells were electroporated with a plasmid-containing wild-type TIM22 (NM_013337) cDNA that had been amplified from HEK293T cDNA and then cloned into the pcDNA5/FRT/TO vector (Invitrogen, V6520-20). Plasmid DNA was electroporated at a final concentration of 250 μg/ml. Electroporation was performed using the Neon™ Transfection system (Invitrogen) using the following conditions: pulse number, 2; pulse width, 20; voltage, 1150. Cells were then immediately resuspended in DMEM medium and incubated at 37°C with 5% CO_2_ for at least 48 h.

### MtDNA genetic studies

MtDNA sequences were obtained by using previously described methods (44,45).

### Whole-exome sequencing, variant calling and bioinformatic filtration

Study participants provided written informed consent for exome sequencing under a protocol approved by the institutional review board of Scripps. DNA was extracted from freshly drawn blood and whole exome sequencing (WES) was pursued utilizing Agilent SureSelect exome hybridization followed by barcoding and sequencing of paired 100 bp reads on an Illumina HiSeq2500 instrument. Read mapping and variant calling and quality filtration was performed using a BWA-GATK best practices variant quality score recalibration approach. Variant annotation was performed using the SG-ADVISER system (46). A series of filters was applied to derive a set of candidate disease causative variants: [1] population-based filtration was liberally set at >1.0% allele frequency in the Exome Aggregation Consortium, 1000 Genomes, National Heart, Lung and Blood Institute (NHLBI) Exome Sequencing Project or Scripps Wellderly populations; [2] functional impact-based filtration to remove variants that are not nonsynonymous, frameshift, inframe, nonsense or do not affect canonical splice-site donor/acceptor sites; and [3] inheritance-based filters to remove variants that do not segregate in the family in a manner consistent with de novo/recessive manner.

### Quantitative PCR

RNA was extracted from control and patient fibroblasts using TRIzol™ reagent (Invitrogen, Carlsbad, California, USA) as per the manufacturer’s instructions. cDNA was generated using the First Strand cDNA Synthesis kit (Thermo Scientific, Waltham, Massachusetts, USA), with random hexamer primers. Quantitative real-time PCR was performed in triplicate using SensiMix SYBR Low-Rox one-step kit and amplification was carried out in a QuantStudio™ 6 Flex System (Applied Biosystems, Foster City, California, USA). Primers for amplification of *TIM22* were 5′-CGGGGAACATCAGACTGGAA-3′ and 5′-GCCTTTAAGCCAGCTCTGAAAC-3′ and levels were normalized against *HPRT* (5′-TGGACAGGACTGAACGTCTT-3′ and 5′-ACAGTCATAGGAATGGATCTATCA-3′).

### Fluorescence microscopy

Fibroblasts were grown on coverslips for at least 24 h. Prior to fixation, cells were incubated with MitoTracker® Orange CMTMRos (Life Technologies) for 15 min and then fixed in 4% paraformaldehyde for 20 min at 37°C. Cells were washed in PBS and mounted in histology mounting medium containing DAPI (Fluoroshield™; Sigma-Aldrich, St.Louis, Missouri, USA). Images were taken using a DeltaVision Spectris epifluorescence microscope (Applied Precision, Issaquah, Washington, USA) at 60× magnification, equipped with a TRITC (excitation 542/27, emission 594/45) and DAPI (excitation 390/18, emission 435/48) filter set. Series of 10–15 sections with 0.5 μm spacing along the Z-axis were taken. Images were deconvoluted and a maximum projection of the stacks was generated by merging the individual slices using the softWORx software (Applied Precision).

### Real-time respirometry

OCR was measured with a XF96 Extracellular Flux Analyzer (Seahorse Bioscience, Billerica, MA, USA). Fibroblasts were seeded the day before the measurement at a density of 40.000 cells/well. Baseline respiration was measured in DMEM supplemented with 1 mm pyruvate and 25 mm glucose after calibration at 37°C in an incubator without CO2. Periodic measurements of oxygen consumption were performed and OCR was calculated from the slope of change in oxygen concentration over time. Metabolic states were measured after subsequent addition of 3 μM oligomycin, 1 μM carbonyl cyanide-4-(trifluoromethoxy)phenylhydrazone, 2 μM antimycin A and 1 μM rotenone.

### Labelling of mitochondrial translation products

*In vivo* radiolabelling in human immortalized fibroblasts and A549 cybrids was done as previously described (47). Cytosolic translation was inhibited with 100 mg/ml emetine and mitochondrial translation pulsed with 0.2 mCi/ml [^35^S]-methionine for 2 h. To visualize radiolabelled proteins, whole-cell lysates were obtained and analysed by SDS-PAGE and autoradiography.

### Enzymatic activities and ATP levels

A quantitative method enzyme-linked immunosorbent assay (ELISA) for cytochrome *c* oxidase specific activity determination was applied using a protocol described elsewhere (48). Citrate synthase (CS) specific activity was measured using previously described protocols (44). For analysis of ATP levels, cells were washed twice with PBS and incubated for 2 h in record buffer containing 5 mm 2-Deoxy-D-glucose and 1 mm pyruvate. Cells were lysed and incubated with luciferin/luciferase reagents (44). All biochemical measurements were done in a NovoStar MBG Labtech (Offenburg, Germany) microplate instrument.

### Mitochondrial isolation and whole-cell lysis

Mitochondria were isolated by differential centrifugation as previously described (8). Protein concentration was measured by Bradford analysis using BSA as a standard.

Human fibroblasts were harvested, pelleted and resuspended in cell lysis buffer [50 mm Tris-HCl (pH 7.5), 130 mm NaCl, 2 mm MgCl2, 1% Nonidet P-40, 1 mm phenylmethylsulfonyl fluoride (PMSF) and protease inhibitor cocktail (Pierce, Waltham, Massachusetts, USA)]. Cell lysates were vortexed briefly and centrifuged at 8000*g* for 5 min at 4°C and the supernatant retained. Cell lysates were incubated with sample dissociation buffer [final concentrations: 6.25 mm Tris-HCl (pH 6.8), 2% SDS, 10% glycerol, 0.01% bromophenol blue and 100 mm DTT] for 30 min at 37°C.

### Miscellaneous

For Blue Native PAGE analyses, isolated mitochondria were solubilized in buffer [0.4% DDM, 20 mm Tris/HCl pH 7.4, 0.1 mm ethylenediaminetetraacetic acid (EDTA), 50 mm NaCl, 10% (w/v) glycerol and 1 mm PMSF] to a final concentration of 1 μg/μl for 20 min at 4°C. Lysates were cleared by centrifugation at 14 000×*g* for 10 min at 4°C and 10× loading dye was added (5% Coomassie brilliant blue G-250, 500 mm 6-aminohexanoic acid, 100 mm Bis-Tris, pH 7.0). Samples were loaded onto 6–16% polyacrylamide gradient gels and separated as previously described (49). SDS/PAGE and western blotting of proteins onto polyvinylidene fluoride membranes (Millipore, Burlington, Massachusetts, USA) was performed using standard methods. Primary antibodies were raised in rabbit or purchased (anti NDUFB8, Abcam, UK; anti SDHB, Abcam; anti UQCRC2, Abcam; anti COXII, Abcam; anti ATP5A, Abcam; anti β-actin, Cloud-Clone Corp., Houston, Texas, USA; anti TIM22, Proteintech, Chicago, Illinois, USA; anti ANT3, Proteintech; anti SDHA, Cell Signalling, Danvers, Massachusetts, USA; anti β-tubulin, Abcam). Antigen**–**antibody complexes were detected using either HRP-coupled secondary antibodies and enhanced chemiluminescence detection on X-ray films (GE Healthcare, Chicago, Illinois, USA), or BioRad, Hercules, California, USA ChemiDoc MP with Image Lab software, or IRDye® 800CW, Li-Cor, Lincoln, Nebraska, USA and detection at 800 nm on a FLA-9000 (Fujifilm, Japan).
